# Contingency Contracts for Weight Gain of Patients with Anorexia Nervosa in Inpatient Therapy: Practice Styles of Specialized Centers

**DOI:** 10.3390/jcm7080215

**Published:** 2018-08-14

**Authors:** Katrin Ziser, Katrin E. Giel, Gaby Resmark, Christoph Nikendei, Hans-Christoph Friederich, Stephan Herpertz, Matthias Rose, Martina de Zwaan, Jörn von Wietersheim, Almut Zeeck, Andreas Dinkel, Markus Burgmer, Bernd Löwe, Carina Sprute, Stephan Zipfel, Florian Junne

**Affiliations:** 1 Department of Psychosomatic Medicine and Psychotherapy, Medical University Hospital Tuebingen, Osianderstr. 5, 72076 Tuebingen, Baden-Wuerttemberg, Germany; katrin.giel@med.uni-tuebingen.de (K.E.G.); gaby.resmark@med.uni-tuebingen.de (G.R.); stephan.zipfel@med.uni-tuebingen.de (S.Z.); florian.junne@med.uni-tuebingen.de (F.J.); 2 Department of General Internal and Psychosomatic Medicine, Heidelberg University Hospital, Im Neuenheimer Feld 410, 69120 Heidelberg, Baden-Wuerttemberg, Germany; christoph.nikendei@med.uni-heidelberg.de; 3 Clinical Institute of Psychosomatic Medicine and Psychotherapy, University Hospital Duesseldorf, Moorenstraße 5, 40225 Duesseldorf, Nordrhein-Westfalen, Germany; hans-christoph.friederich@med.uni-duesseldorf.de; 4 Department of Psychosomatic Medicine and Psychotherapy, LWL University Hospital, Ruhr-University Bochum, Alexandrinenstr. 1-3, 44791 Bochum, Nordrhein-Westfalen, Germany; stephan.herpertz@rub.de; 5 Division of Psychosomatic Medicine, Charité University Hospital Berlin, Hindenburgdamm 30, 12200 Berlin, Berlin, Germany; rose@charite.de; 6 Department of Psychosomatic Medicine and Psychotherapy, Hannover Medical School, Carl-Neuberg-Straße 1, 30625 Hannover, Niedersachsen, Germany; dezwaan.martina@mh-hannover.de; 7 Department of Psychosomatic Medicine and Psychotherapy, University Hospital Ulm, Albert-Einstein-Allee 23, 89081 Ulm, Baden-Wuerttemberg, Germany; joern.vonwietersheim@uniklinik-ulm.de; 8 Department of Psychosomatic Medicine and Psychotherapy, University Hospital Freiburg, Hauptstr. 8, 79104 Freiburg, Baden-Wuerttemberg, Germany; almut.zeeck@uniklinik-freiburg.de; 9 Department of Psychosomatic Medicine and Psychotherapy, Klinikum rechts der Isar, Technical University of Munich, Langerstr. 3, 81675 Munich, Bayern, Germany; a.dinkel@tum.de; 10 Department of Psychosomatic Medicine and Psychotherapy, University Hospital Muenster, Domagkstr. 22, 48149 Muenster, Nordrhein-Westfalen, Germany; markus.burgmer@ukmuenster.de; 11 Institute and Outpatient Clinic for Psychosomatic Medicine and Psychotherapy, University Hospital Hamburg-Eppendorf, Martinistraße 52, 20246 Hamburg-Eppendorf, Hamburg, Germany; b.loewe@uke.de; 12 Department of Psychosomatic Medicine and Psychotherapy, LVR-University Hospital, University Duisburg-Essen, Virchowstr. 174, 45147 Essen, Nordrhein-Westfalen, Germany; carina.sprute@lvr.de

**Keywords:** Anorexia nervosa, treatment contracts, weight gain, inpatient treatment, survey

## Abstract

The treatment of patients with anorexia nervosa (AN) is often challenging, due to a high degree of ambivalence towards recovery and weight gain these patients often express. One part of the multimodal treatment is the utilization of treatment contracts (i.e., contingency contracts) that aim to motivate patients to gain weight by applying positive and negative consequences for the (non-)achievement of weight goals. The main aim of this study is to assess and analyze current standards of contingency contracts’ utilization in German eating disorder centers. *n* = 76 mental health professionals of twelve specialized university centers in Germany that are currently or were formerly treating patients with AN in an inpatient setting participated. Most experts use contingency contracts in their clinic with weekly weight goals ranging between 500 and 700 g. Overall effectiveness and significance of contingency contracts for the inpatient treatment of patients with AN was rated high. Typical characteristics of a contingency contract in specialized German university hospital centers, such as the most frequent consequences, are described. The survey results assist the planning of further studies aiming to improve the multimodal treatment of patients with AN. For clinical practice, using external motivators such as contingency contracts as well as targeting internal motivation (e.g., by using motivational interviewing) is proposed.

## 1. Introduction

According to the Diagnostic and Statistical Manual of Mental Disorders (DSM-5), anorexia nervosa (AN) is a mental disorder characterized by an intense fear of gaining weight and body image disturbances that lead to restricted food intake relative to the required food intake (restrictive subtype) or other behaviors promoting weight loss such as excessive exercising or purging behavior (binge-purge subtype) [1]. Despite a twelve-month prevalence rate of 0.8% within the German population, AN is one of the mental disorders with the highest mortality [2,3,4]. In the long term, only approximately half of patients completely recover, whereas roughly 20 percent develop a chronic form of the disorder [5,6]. It takes an average of five to six years to achieve complete recovery [2]. This reflects the challenges associated with treatment, resulting from a high degree of ambivalence towards recovery and weight gain patients with AN often express [7,8].

According to German treatment recommendations, patients with severe AN (Body Mass Index (BMI) < 15) are treated in inpatient settings that regard weight restoration as one of the focal points for recovery [9]. Initial weight gain and symptom-orientation have been shown to predict good outcomes [10,11]. Nonetheless, a study by Schlegl and colleagues suggests that about one third of patients with AN do not show a significant response to intensive inpatient treatment [12]. Thus, there is still room for improvement in inpatient treatment approaches for patients with AN [13]. 

One indispensable part of the multimodal treatment approach for patients with AN is the utilization of a treatment contract, which is implemented to induce motivation for weight gain. Treatment contracts are currently routinely used in the inpatient treatment of patients with AN in Germany [14,15,16,17]. They are verbal or written agreements with the patient that contain mostly, but not exclusively, weight goals. Frequently, they outline the amount of weight that should be gained in a defined period of time during the inpatient stay (e.g., each week). Positive consequences for reaching these weight goals and negative consequences for not fulfilling weight goals are determined. Treatment contracts to induce weight gain in patients with AN can also be called weight contracts or contingency contracts for weight gain. 

A recent systematic review by our group showed that despite their routine usage in inpatient treatment, contingency contracts for weight gain are an understudied topic and the empirical evidence base is scarce [18]. The majority of publications included in our review were of rather historical nature with few current contributions. We could, however, identify a development from restrictive applications of treatment contracts, e.g., in the form of bed rest to more collaborative approaches. These collaborative approaches try to actively involve patients into the contingency contracting process, e.g., by negotiating terms of the contract or letting patients choose consequences. 

In terms of clinical application, there is some guidance in available treatment manuals for AN [15,19] with written examples of contracts. However, this guidance seems to stem from clinical expertise which is valuable but not sufficient to ensure high treatment standards. Currently, it is unclear whether treatment manuals are used and if done so, how the contingency contract process is organized in German eating disorder centers.

The main aim of the present study is to assess and analyze the utilization of contingency contracts for weight gain in German university hospitals specializing in eating disorders by means of a survey. Current approaches used by these specialized centers for collaboration with the patient are investigated, as well as strategies to enhance the patients’ autonomy and motivation in the treatment contract process. Experiences of mental health professionals during the treatment contract process are also described. Finally, as an exploratory question, the role of professional characteristics in the contingency contract process is examined. 

## 2. Experimental Section

### 2.1. Study Centers

Twelve specialized university hospital centers in Germany were invited and participated in this multicenter study. Mental health professionals who are currently or were formerly treating patients with AN in an inpatient setting were eligible for participation.

### 2.2. Sample

The study sample consists of *n* = 76 medical doctors and clinical psychologists between the ages of 24 and 60 (*M* = 37.95, *SD* = 8.28). For a detailed sample description, see Table 1. 

### 2.3. Measures

The online survey contained questions concerning the following topics: (1) demographic characteristics (including e.g., therapeutic orientation and clinical work experience); (2) questions about the utilization of and criteria for implementing contingency contracts into the inpatient treatment routine (e.g., percentage of patients that receive a contingency contract, timepoint of conclusion, duration, standardization of the procedure); (3) precise form of the contingency contract (e.g., verbal, written, freely formulated); (4) weight goals, control days and consequences for achieving or not achieving the weight goals; (5) circumstances that lead to a termination of contingency contracts; (6) experienced emotions of experts during the contingency contract process and appraisal of effectiveness. Items were either dichotomous (applicable–not applicable) or measured on a seven-point Likert scale (e.g., 1 never–7 always). All items relating to the contingency contract were newly developed for this survey.

After the demographic questions, a definition of contingency contracts in the context of inpatient treatment of patients with AN was given for clarification. Contingency contracts in the form of a weight contract were defined as follows: “A weight contract is a verbal or written agreement with a patient that determines weight changes and/or behavioral changes (e.g., eating behavior) that are linked to consequences for the patient.”

### 2.4. Procedure

Invitational links were sent to representatives of all of the study centers, who then forwarded the invitation to eligible expert staff. Upon clicking on the survey link, experts were informed about the survey and protection of data privacy. They had to give consent in order to start the survey. Upon reaching the final page, experts were informed that the survey is finished and thanked for their participation.

### 2.5. Statistical Analyses

Means, standard deviations and percentages are reported for sample descriptions. Since variables were mostly not normally distributed, Mann–Whitney *U* tests for the analyses of single differences of means were used. To analyze potential associations between variables, Spearman rho correlations are reported. All statistical analyses were performed using IBM SPSS Statistics version 24 (IBM Corporation, Armonk, NY, USA). The level of significance for all analyses was set at *α* = 0.05.

## 3. Results

### 3.1. Utilization

All of the experts reported that contingency contracts are utilized in their institution for inpatients with AN. They estimated that 87.6 percent of patients with AN in their department and 87.9 percent of their own patients with AN receive a contingency contract (annualized rate). The majority of experts (90.8%) reported using a standardized procedure to put contingency contracts in place. Of those, almost all reported having a guideline/manual provided within the department (98.6%) versus e.g., a published manual.

### 3.2. Preparation and Conclusion

About two thirds of experts (65.8%) reported preparing contingency contracts before inpatient treatment, for example at a preliminary (outpatient) consultation. 14.5 percent of the experts reported on giving written information about contingency contracts to the patient before admission to the ward. The majority of experts (78.4%) reported that contingency contracts are finalized in the first week of the inpatient stay. Only 5.2 percent of experts reported finalizing contingency contracts in the second week of inpatient treatment and 5.3 percent reported on not having a set time point for concluding contingency contracts.

### 3.3. Weight Contingencies and Weight Goals 

Most experts (88.2%) reported setting standardized weekly contingents for weight gain, ranging between 300 g and 800 g per week. The most frequent weight gain goals are 700 g per week (44.8%), 500 g per week (37.3%) and 800 g per week (10.4%). The determination of the designated weight goal differs between the institutions: 46.1 percent of experts indicated individually negotiating the weight goal with the patients, and 39.5 percent of experts indicated that the weight goal is orientated at normal or close to normal weight with BMIs ranging between 17 and 19 kg/m^2^. One expert indicated using different BMIs according to the age group of the patient for determining normal weight. For 15.8 percent of experts, weight goals were adapted to the planned duration of treatment. Only 6.6 percent of experts reported on not having a determined weight goal.

### 3.4. Revisiting, Changing and Terminating Contingency Contracts 

Experts reported on revisiting the contingency contract with the patients at determined time points (47.4%), predominantly during ward rounds. Some other cases, e.g., if weight loss occurred, also made it necessary to revisit contingency contracts. For a smaller proportion of experts, revisiting the contingency contract occurred routinely after weighing the patient (28.9%), in the event of negative consequences (28.9%) or in the event of positive consequences (25.0%). Only 27.6 percent of experts reported on revisiting the contingency contract in each session.

Changing contingency contracts in the course of the inpatient treatment seemed to be handled quite differently: About one third of the experts (32.9%) reported on changing contingency contracts when patients lost weight and/or dropped below a certain BMI or when patients could not catch up to the required amount of weight gain anymore (31.6%). Some experts (7.8%) reported that changes/adaptions of the contingency contract were not intended whereas other experts reported on individually adapting contingency contracts over the course of treatment. Individually adapting might for instance take the form of temporarily changing from weight gain to weight maintenance. Some experts also reported on discharging patients from the ward for motivational reasons if weight goals were repeatedly not achieved. Patients were then offered the possibility of a re-admission after one or two weeks if they achieved some weight gain on their own. 

About half of the experts (48.6%) reported that terminating contingency contracts did not happen in their institution, whereas 39.2 percent indicated that contingency contracts were terminated in special cases. These include the achievement of normal weight, somatic reasons (e.g., refeeding syndrome, infections) or if other symptoms gain priority (e.g., impulsive behavior). 

### 3.5. Consequences

Consequences mostly depended on weight loss (90.8%) and weight gain (86.8%). One quarter of experts also reported that consequences could depend on symptoms like vomiting/purging, exercising/physical activity and eating behavior. Consequences were routinely applied after checking weight, either after every weighing (31.6%) or every second weighing (47.4%). Experts reported on choosing positive consequences themselves (23.7%) or letting the patient choose positive consequences from a list (17.1%) or freely (26.3%). In about a quarter of cases (24.7%), consequences were already determined in the contingency contract or were negotiated with the patient (13.0%). In regards to negative consequences, 36.8% experts reported determining the consequences themselves, as opposed to letting patients choose from a list (21.1%) or freely (11.8%). 

Most frequently used positive consequences were the cessation of ward restriction (84.2%), being able to temporarily leave the hospital (82.9%) and the cessation of a liquid diet. Other mentioned positive consequences were: extension of treatment opportunities (e.g., patients could also participate in art or music therapy), cessation of accompanied eating, cessation of nasogastric feeding, and opportunities to temporarily leave the ward. When patients could choose their own positive consequences, chosen consequences included: buying themselves something nice, having their hair done, having a meal outside of the hospital, meeting friends, taking a bath, watching a movie/going to the cinema, bringing one’s musical instrument to the ward and using the music room.

The most frequently used negative consequences were restriction to the ward (86.8%) and additional high caloric nutrients (69.7%). Further mentioned negative consequences were movement bans, nasogastric feeding, closely accompanied eating, and restrictions on using the phone or having visitors. The ultimate negative consequence was discharge from the hospital. 

### 3.6. Overall Effectiveness and Factors of Success from the Experts’ Points of View

Overall effectiveness of contingency contracts in the inpatient treatment of patients with AN was rated as ‘effective for the most part’ (*M* = 5.72, *SD* = 0.74). Greater clinical work experience was associated with a higher appraisal of the relevance of contingency contracts for the inpatient treatment of patients with AN (*r_s_* = 0.328, *p* = 0.006).

**Figure 1:**
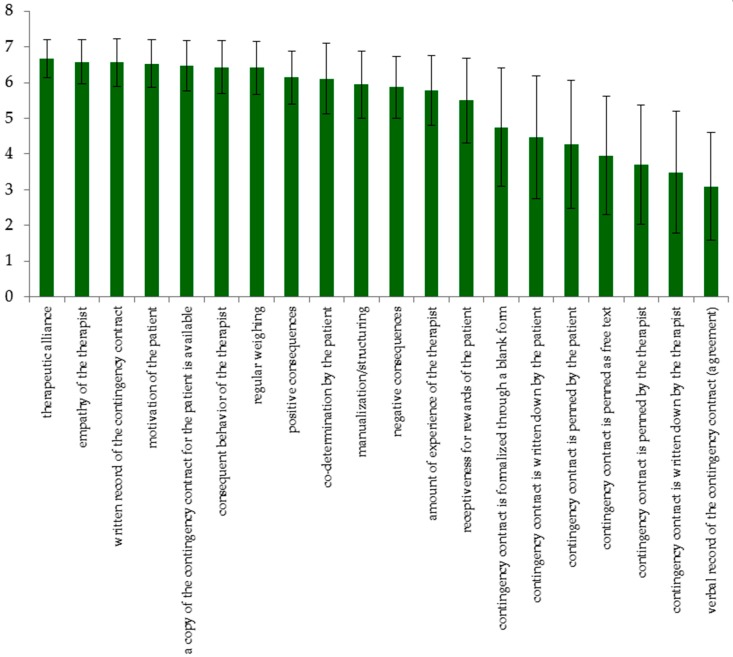
Factors of success of contingency contracts for anorexia nervosa (AN) by expert ratings. Factors of success were rated on a 7-point Likert scale from 1 ‘not important at all’ to 7 ‘very important’.

Among the factors experts rated as important for the success of a contingency contract were general factors such as therapeutic alliance (*M* = 6.67, *SD* = 0.53), empathy of the therapist (*M* = 6.58, *SD* = 0.62) and motivation of the patient (*M* = 6.53, *SD* = 0.67). Factors such as having a written record of the contingency contract (*M* = 6.56, *SD* = 0.67) and having a copy of the contingency contract available for the patient (*M* = 6.47, *SD* = 0.71) were also rated as important. For a detailed rating of factors of success, see Figure 1.

### 3.7. Emotions Experienced by the Experts during the Contingency Contract Negotiation and Emotional Burden

On average, experts rated the overall significance of contingency contracts for the inpatient treatment of patients with AN as ‘significant for the most part’ to ‘very significant’ (*M* = 6.27, *SD* = 1.00). They reported on not experiencing the contingency contract process (preparation, negotiation and conclusion) as emotionally straining (*M* = 3.95, *SD* = 1.65), however there was a significant correlation between experiencing emotional strain and the amount of clinical work experience in years of *r_s_* = −0.355, *p* = 0.002. This indicates that when clinical work experience increases, emotional strain during the contingency contract process decreases. 

Experts reported mainly experiencing a sense of responsibility (*M* = 5.20, *SD* = 1.09), compassion (*M* = 4.80, *SD* = 1.09) and strain (*M* = 4.54, *SD* = 1.20) during the negotiation of contingency contracts. Other emotions (tension, relaxation, ambivalence, frustration, anger and rejection) were reported as being experienced ‘rarely’ to ‘occasionally’.

### 3.8. Group Differences

Potential differences in appraising contingency contracts in the inpatient treatment of patients with AN were tested between occupational groups (medical doctors vs. psychologists) and between therapeutic orientations (behavior therapy vs. psychodynamic therapy). Regarding differences between occupational groups, there were no significant differences in emotions experienced during the negotiation of contingency contracts (all *U*s > 406.50, all *p*s > 0.171). However, medical doctors rated the ethical tenability of contingency contracts, especially regarding the application of negative consequences such as restriction to the ward, higher than psychologists (*U* = 373.50, *p* = 0.008). 

Regarding the emotions experienced during the contingency contract process, differences in how ambivalence was experienced were found between therapeutic orientations (*U* = 314.00, *p* = 0.060). Specifically, psychodynamic therapists experienced more ambivalence while negotiating a contingency contract. There were no group differences for the other listed emotions (all *U*s > 362.00, all *p*s > 0.302). For the ratings of factors of success, there was only one significant difference between the therapeutic orientations: Behavioral therapists rated recording the contingency contract in a written form as more important compared to psychodynamic therapists (*U* = 359.00, *p* = 0.019).

## 4. Discussion

This study analyzed characteristics, utilization and appraisal of contingency contracts for weight gain in AN in German university hospitals specializing in the treatment of eating disorders. Experts were asked about their preparation, negotiation, conclusion and revisions of contingency contracts for patients with AN, their overall rating of effectiveness, as well as experienced emotions and possible emotional strain during this process. 

### 4.1. Similarities of Contingency Contracts in Specialized Eating Disorder Centers

As expected, the majority of patients with AN receive a contingency contract in the participating institutions. Although not following a published manual, utilization in all centers follows internal guidelines or manuals. The most commonly used weight goals range between 500 and 800 g per week and are therefore in line with current recommendations of treatment guidelines for eating disorders [9,20].

Consequences are usually dependent on weight gain and/or weight loss. Only a few experts reported also putting consequences on other eating disorder related behaviors such as excessive exercising or vomiting. Having weight gain or weight loss as a sole focus of contingency contracts for patients with AN presumably originates from early behavioristic approaches of contingency management [18]. In light of a holistic treatment approach however, it seems advisable to consider other eating disorder related behaviors such as excessive exercising or vomiting as part of the contingency contract as well. 

**Figure 2:**
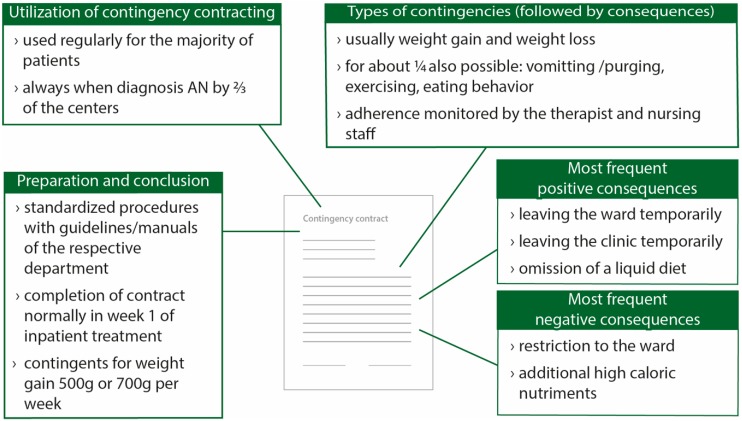
Characteristics of typical contingency contracts in specialized German university centers; AN = anorexia nervosa.

In sum, the present study uncovered basic characteristics of contingency contracts shared by the majority of experts. A typical contingency contract in specialized eating disorder centers in Germany can therefore be described as presented in Figure 2.

### 4.2. Differences of Contingency Contracts in Specialized Eating Disorder Centers

The three major aspects, in which participating institutions differ, are their definitions of weight goals, when and how contingency contracts are revisited and the choice of consequences. The definition of weight goals and the revisiting of contingency contracts possibly reflect the different self-developed manuals of the eating disorder centers. One half of experts negotiate weight goals with the patient, also taking aspects such as planned duration of stay into consideration. Another 40% orientate themselves toward a BMI value that should be achieved (low normal). One possible explanation for different BMI goals, ranging between 17 and 19 kg/m^2^, is the continued discussion surrounding which BMI cut-off should be used to indicate non-anorectic weight for patients with AN [1,21].

Herzog and colleagues [22] showed that lower weekly weight goals (500 g) led to a higher achieved weight at the end of treatment, compared to higher weekly weight goals (750 g). In contrast, there are studies showing that higher weekly caloric intake led to higher overall weight gain (e.g., [23]). A recent systematic review [24] demonstrated that higher calorie refeeding is not associated with increased risk of the refeeding syndrome, at least for mildly and moderately affected patients. However, inpatient therapy is mainly indicated for severely ill patients and caution regarding caloric intake in the first days of treatment should be applied. Hence, for severely malnourished patients, there is no evidence to change current approaches. However, the long-term impact of different approaches is unknown [24], therefore no clear recommendation can be made from the literature concerning what (weekly) weight goals or BMI goals should be set [9].

### 4.3. Collaborative Approaches within the Contingency Contract Process 

One of the aims of the current study was to identify approaches to incorporate patients into the contingency contract process in order to motivate them and enhance compliance and autonomy. In concrete terms, patients (and parents for younger patients) can for example co-determine weight goals, choose consequences and write down the contingency contract in their own words. We found that about 30% of experts let their patients choose consequences or negotiate consequences with the patient and almost half of the experts negotiate weight goals with their patients. This seems promising especially considering that patients are often ambivalent to restore weight [25]. However, letting patients co-determine weight goals can bare the risk of setting weight goals that are too small. Additionally, negotiating weight goals and weight contingencies with the patient instead of setting them oneself can be a wearisome task for the therapist. 

In the literature covering the topic of collaboration in the field of AN, there is a clear preference for collaborative approaches by patients as well as therapists [26]. Furthermore, Williams and Reid [25] showed in their qualitative study that patients with AN feel low self-efficacy about changing behaviors. On one side, AN gives them a sense of control, but the disorder also causes strong feelings of loss of control. The authors conclude that this ambivalence patients experience should be targeted in a collaborative manner [25]. In line with this evidence and the systematic review, which shows an overall movement from the utilization of (directive) contingency management towards more collaborative contingency contracts [18], contingency contracts in Germany should intensify their focus on collaboration within the weight contract process. 

### 4.4. Implications for Clinical Practice 

Motivational aspects are one of the main considerations for the improvement of treatment and care for patients with AN [27] and are taken into consideration for example by integrating motivation-based therapeutic styles into treatment [28,29]. Motivation of patients has been shown to play an essential role concerning dropout rates and treatment compliance [30]. Furthermore, internal motivation to change was identified as one of the positive predictors of clinically significant changes in eating disorder psychopathology in patients with AN quantitatively [12] as well as qualitatively in patients’ reports [31]. It is therefore worth considering a shift in the treatment approach, from motivating the patients externally through a contingency contract, to enhancing internal motivation for example by incorporating a motivations-based treatment style. Indeed, some of the authors are currently developing a motivation-based intervention, incorporating motivational aspects into the inpatient treatment of patients with AN, which they plan to soon test against existing treatment options. 

### 4.5. Experiences of Experts with the Contingency Contract Process

Although there was great variation in emotions experienced by the participating experts during the contingency contract process, on average there was no extremely negative or positive emotions which evidently accompanied this process. Additionally, it could be shown that the emotional burden for experts decreased with the years of experience, which coincides with clinical impressions. We found no significant differences between occupational groups (physician versus psychologist) or therapeutic orientation (behavioral versus psychodynamic psychotherapy). It would be interesting, however, to also investigate the emotional experiences of nurses and other caregivers in the inpatient environment. 

### 4.6. Limitations and Prospects

The main limitation of the study is its descriptive nature; as no causal conclusions about the reported associations and group differences can be drawn. Furthermore, only specialized university centers were included which heightened the risk of a selection/recruitment bias. Although a fair amount of specialized university centers in Germany participated, not all could be included, raising the question of representativeness. However, given that this is the first study attempting to describe the contingency contract process in specialized centers in Germany, this account provides a valuable contribution to the evidence base. 

The usefulness of contingency contracting practice styles for patients with AN in other countries would also be of high interest. Taking into account that patients in other countries might be treated in outpatient or day-patient settings more frequently, it might be more difficult to track positive and negative consequences. However, this would also entail the chance for greater collaboration between patients and therapists, potentially lessening the chance of patients fearing ‘to be controlled’ and enabling them to gain more autonomy. There are some reports about the utilization of contingency contracts around the world [18], however to the best of our knowledge, studies about practice styles that go beyond the treatment program of one specific clinic, are missing.

## 5. Conclusions

The majority of experts use contingency contracts for their patients with AN. Most contracts involve weekly weight goals and there is strong consensus regarding the most frequent positive and negative consequences following weight gain or loss. This knowledge can help define and implement best practices concerning contingency contracting in the treatment of patients with AN. It also gives insight into current practice, and is therefore useful for the planning of further studies aiming to improve the efficacy of treatments procedures for patients with AN. For clinical practice, using external motivators such as contingency contracts as well as targeting internal motivation (e.g., by using motivational interviewing) is proposed.

## Figures and Tables

**Table 1:** Demographic characteristics of the study sample (*n* = 76).

Variables	*M* (*SD*)	%
Gender: female		71.1
Clinical experience in psychotherapy/psychosomatic medicine/psychiatry in years	7.75 (7.20)	
Occupational group		
Medical doctor		61.8
Clinical psychologist		36.8
Both		1.3
Estimated number of treated patients with anorexia nervosa		
<20		34.2
20–40		23.7
41–60		14.5
61–80		6.6
81–100		9.2
>100		11.8
Main therapeutic orientation		
Cognitive-behavior psychotherapy		29.7
Psychodynamic psychotherapy		70.3
